# Experience and views of primary care physicians involved in reorganisation of care in family medicine practices during COVID-19 pandemic: A qualitative study from Slovenia

**DOI:** 10.1080/13814788.2023.2193886

**Published:** 2023-04-04

**Authors:** Simona Kovačec, Zalika Klemenc-Ketiš, Antonija Poplas-Susič, Andrej Kravos

**Affiliations:** a Department of Family Medicine, Faculty of Medicine, University of Maribor, Maribor, Slovenia; b Community Health Centre dr. Adolfa Drolca Maribor, Maribor, Slovenia; c Community Health Centre Ljubljana, Ljubljana, Slovenia; d Department of Family Medicine, Faculty of Medicine, University of Ljubljana, Ljubljana, Slovenia

**Keywords:** Primary care, COVID-19 pandemic, adaptation of health system, decision-making institution

## Abstract

**Background:**

In Slovenia, primary care is the backbone and gatekeeper to the health care system. During the first months of the COVID-19 pandemic, primary care had to be reorganised to manage suspected COVID-19 patients, safely care for other patients, and manage the consequences of the pandemic.

**Objectives:**

To explore the opinions and experiences of Slovenian primary care workers (PCWs) about their confrontation with COVID-19.

**Methods:**

In June 2020, we conducted a qualitative study among PCWs in Slovenia. Invited participants (*n* = 42) worked either in primary health care centres or as private contractors and were involved in organising care during the COVID-19 pandemic. The study was conducted using semi-structured online questionnaires. Data was analysed using an inductive-deductive method.

**Results:**

Out of 42 invited subjects, 18 participated in the study. The main predefined categories were Information/from decision-makers, Organisation of work, Workforce, Personal protective equipment, Views on decision-making institutions, Stressors that place additional burden on health workers, and Suggestions for improvement (funding, organisation of care). Within these categories, 29 themes emerged.

**Conclusion:**

Based on participants’ experiences and suggestions, the most important areas to address in similar pandemic situations are clear organisation of work in primary care (adequate funding, staff allocation, distribution of personal protective equipment), psychological solid support for health workers, and effective and timely support from health authorities.

KEY MESSAGES

- Primary care workers in Slovenia felt that establishing administrative practices, separate practices for non-COVID and COVID patients, and entry points for swabs would ensure a sustainable health care system.
- Primary care workers in Slovenia experienced a lack of support from health authorities and had to organise themselves.

## Introduction

COVID-19 pandemic profoundly impacted all spheres of life, including health care (HC) around the world and in Slovenia [1]. On 4 March 2020, the first infection with COVID-19 was confirmed in Slovenia [2]. Slovenia declared the epidemic on 12 March 2020. As in many other countries, the Slovenian healthcare system was unprepared for the pandemic [3]. Primary health care had to be reorganised during the pandemic to cope with suspected cases of COVID-19, other patients (e.g. chronically ill), and the consequences of the pandemic [3,4].

Slovenia has a statutory health insurance system with a single public insurer providing universal health services coverage. Primary health care (PHC) is based on a network of community primary health centres (CPHC) organised by municipalities and private physicians, who are concessionaires, and included in the national primary health care system. In Slovenia, there are 63 CPHCs organised in 10 administrative regions. Three CPHCs have more than 50 primary care teams, and about one-third of CPHCs are small, with up to five teams. Slovenia has about 900 PHC teams, of which about 200 are privately operated with a concession.

During the pandemic, the Ministry of Health (MH) decided on priorities for medical care at the national level, developed regulations and instructions, and organised the necessary equipment at the national level [2]. The Slovenian Medical Chamber (SMC) organised training and provided important technical and organisational information [5].

This study aimed to investigate the opinions and experiences of PHC staff on the reorganisation of care during the COVID-19 pandemic in Slovenia. The Slovenian model of primary care reorganisation during COVID-19 was one of the many models in Europe [6]. We wanted to identify the advantages and limitations of this model to propose improvements to be better prepared for future health crises and to offer the international community to learn from the Slovenian experience.

## Methods

### Design of the study

This study was an inductive-deductive qualitative study.

### Study population

We targeted participants who were PHC staff in Slovenia, either in community primary health care centres or as private contractors, and who were involved in restructuring care during the pandemic as managers, department heads, members of professional associations, etcetera. All participants were required to give informed consent to participate in the study.

We used a purposive sampling method. Invitations to participate were sent to 63 CPHCs in Slovenia and to 202 private contractors. We asked CPHCs (*n* = 63) and private contractors (*n* = 202) to identify individuals who met the inclusion criteria. The same question was also sent to the Slovenian Departments of Family Medicine and the Slovenian Association of Family Medicine. In this way, 42 individuals from all administrative regions were identified. They all knew the researchers personally but were not informed about the start of the study before it began.

### Data collection

Data collection was conducted in June 2020 using a semi-structured online questionnaire. The questions were entered into Google Docs to create an electronic questionnaire version. The link to the questionnaire was sent to participants’ email addresses on 1 June 2020. A reminder was sent on 15 June 2020.

### Questionnaire construction

Quantitative data was collected on age, gender, education, occupation and job, service length and facility location (rural, semi-urban, urban). If a participant worked in a village or settlement, this was described as a rural area. A semi-rural area was defined as a small town. An urban area was defined as a large city.

Qualitative data were collected through open-ended questions. The authors asked the open-ended questions under seven predefined thematic categories: Information/instructions from decisionmakers, Organisation of work, Workforce, Personal protective equipment (PPE), Views on decision-making institutions, Stressors that place additional burden on health workers, and Suggestions for improvement. The questions were based on the literature, and the questionnaire was piloted with seven PHC experts (professors of family medicine, heads of primary care professional associations, CPHC directors) in Slovenia [7,8]. They were asked to evaluate the questions’ comprehensibility, report any problems in answering the questions, give their opinion on the relevance of the existing questions and suggest deletion or addition of questions.

### Data analysis

Responses to the open-ended questions in each category were analysed using an inductive-deductive method. Two researchers (family medicine specialists, senior researchers in family medicine and experts in qualitative methodology) analysed the text independently. The supervisor was an expert in qualitative analysis and an expert in the field of research (PHC - family medicine). He was consulted in case of disagreements between the two researchers to harmonise opinions. The researchers established different codes during the analysis, grouping them into thematic sections under a certain predefined category.

## Results

### Demographic characteristics

Of the forty-two individuals who met the inclusion criteria, eighteen participated in the study. Participants were from eight of ten administrative regions. Most (15/18) of the participants were family physicians (one gynaecologist, one paediatrician, one registered nurse), and 67 percent were female (Table 1).

**Table 1.** Demographic and professional data of participants (*n* = 18).

Characteristics		Mean (SD)
Age		48 (9)
Length of service		21 (9)
		Number (%)
Gender	Male	6 (33)
	Female	12 (67)
Location of institution	Urban	12 (67)
	Semi-urban	3 (17)
	Rural	3 (17)
Education^a^	Specialisation	17 (94)
	Master’s degree or PhD	6 (3)
Profession	Family physician	15 (82)
	Registered nurse	1 (6)
	Paediatrician	1 (6)
	Gynaecologist	1 (6)
Employment	Public health centre	13 (72)
	Private within public system	4 (22)
	Private health centre	1 (6)
Leadership function	Director^b^	3 (17)
	Professional director	6 (33)
	Head of the Department of Family Medicine	4 (22)
	Head of a unit in CHPC	5 (28)

^a^The numbers do not add as one person can have both specialisation and master’s degree or PhD.

^b^General manager of the CPHC, leads the CHPC.

### Categories and themes

We discuss the results using the seven predefined categories (Table 2).

**Table 2.** Categories and themes.

Category	Theme
Information/instructions from decision-makers	Timeliness of instructions Clarity and accuracy of instructions Consistency of the content of instructions between different institutions Instruction brought additional administrative burdens
Organisation of work	Establishment of an entry point Establishment of a practice for COVID patients Regional coverage Centralisation of other activities Practices for non-COVID patients Administrative practices
Workforce	Staff adequacy Appropriate staff training Involvement of external collaborators
Personal protective equipment (PPE)	Insufficiency / lack of PPE Ingenuity in providing PPE The role of the MH in the procurement of PPE
Experiences with the decision-making institutions	Slovenian Medical Chamber National Health Insurance Institute Professional bodies Nurses and Midwives Association of Slovenia Ministry of Health National Institute for Public Health
Stressors that place an additional burden on healthcare workers	Patients’ demands Rapid changing of instructions Lack of professional information Fear Uncertainty
Suggestions for improvements	Financing Organisation

#### Information/instructions from decision-makers

Participants indicated that instructions were not timely, often unclear and differed between the institutions (many participants mentioned this).

*Too many notifications at the same time, too fast changes of instructions, decisions from today to tomorrow. (Female, 43 years old, family physician, CPHC, professional director)*

*Too frequent instruction changes and too many administrative burdens in the PHC. (Female, 41 years old, family physician, CPHC, head of the department)*

#### Organisation of work

Participants pointed out that PHCs are organised to prevent the spread of infection and that activities were centralised. However, organisation was left to individual primary care providers (many participants mentioned this).

*We had to organise ourselves. (Female, 40 years old, family physician, CPHC, professional director)*

*Practices for infected patients were separated from non-infected patients; they had separate access and different staff which after a week of work in the practice went home for 2 weeks off. (Female, 43 years old, family physician, CPHC, professional director)*

*We centralised all activities from the point of view of optimal distribution of staff and ensuring the sustainability of the health care system in the event of more infected patients and more infected health care personnel as well from the point of view of optimal use of PPE. (Female, 41 years old, family physician, CPHC, professional director)*

#### Workforce

Participants pointed out the lack of staff in general and the lack of properly trained staff (many participants mentioned this).

*With the opening of other activities and the beginning of training there was a lack of staff. (Male, 50 years old, family physician, CPHC, professional director)*

*We additionally started with overtime work; the lack of staff was substantial. (Female, 41 years old, family physician, CPHC, professional director)*

*We trained mainly nurses to ensure the most optimal swabbing to save doctors. (Female, 52 years old, nurse, CPHC, head of a unit)*

*Private doctors were differently prepared to be involved in the work at entry points: some yes, others no. We had problems due to different legal opinions on contracts between CPHC and private doctors. (Female, 41 years old, family physician, CPHC, professional director)*

#### Personal protective equipment

At the beginning of the pandemic, there was a shortage of PPE (many participants mentioned this). This was especially true for private providers.

*As a private doctor, I could not get any PPE in the first month.* *Permanent suppliers had nothing, and we were looking for new suppliers from whom we were buying. (Female, 55 years old, private, head of unit)*

*They (MH) didn’t even answer (even though they established a dedicated email address intended for communication due to the pandemic) about what and when we can get or order. (Female, 55 years old, private, head of unit)*

*We were without impact on what we will get, when we will get it and without information on how it will be in future. (Male, 50 years old, family physician, CPHC, professional director)*

#### Experiences with the decision-making institutions

Slovenian Medical Chamber (SMC) sent helpful information (some participants mentioned this).

*SMC had a more supportive role. It sent especially useful emails that summarised all the daily news from all participants (Ministry of Health (MH), National Health Insurance Institute (NHII). (Male, 53 years old, private, head of a unit)*

The NHII responded slowly and ambiguously. They reduced some of the administrative burdens on practices but also introduced some additional burdens (many participants mentioned this).

*Too slow reaction and a lot of ambiguity. (Male, 53 years old, private, head of unit)*

*The fact that (NHII) decided that we can provide services even without the presence of a health insurance card was an advantage. (Female, 41 years old, family physician, CPHC, professional director)*

The Nurses and Midwives Association of Slovenia (NMAS) was evaluated as very constructive in organising care in nursing homes.

*The NMAS was very constructive, especially in the issue of the organisation of health care in nursing homes. (Female, 52 years old, nurse, CPHC, head of unit)*

MH was not ready for the pandemic.

*People who prepared instructions were sometimes disoriented and without contact with reality.’ (Female, 55 years old, private, head of unit)*

*The MH is not ready for such a situation. There seems to be no national plan for dealing with a crisis as decisions have been adjusted continuously. (Female, 55 years old, private, head of unit)*

NIPH took some time to organise and improve the clarity of information for professionals and the public. Instructions changed quickly and were often contradictory (many participants mentioned this).

*Their instructions were delayed in the initial phase. (Female, 55 years old, private, head of unit)*

*The answers of epidemiologists from regional units varied and were very subjective. (Female, 43 years old, CHPC, professional director)*

#### Stressors that place an additional burden on healthcare workers (many participants mentioned this)

Participants pointed to the high demands of patients, rapid changes in instructions, lack of professional information, and fear of health care workers. However, psychological support was available.

*Uncertainty, patient’s intolerance, poor cooperation with the secondary level. (Female, 40 years old, family physician, CPHC, professional director)*

*Uncertainty, unclear funding, staff exposure. (Female, 41 years old, family physician, CPHC, professional director)*

*Waiting every day for the next day, expecting the worst. (Male, 50 years old, CPHC, director)*

*The psychologists and clinical psychologists were available by phone and email to both staff and patients, and all staff in psychological distress had access to consultations with external psychiatrists. ((Female, 41 years old, family physician, CPHC, professional director)*

#### Suggestions for improvement

According to participants’ suggestions, the most critical areas to address in similar pandemic situations are a clear organisation of primary care work (adequate and transparent funding, staff allocation, distribution of personal protective equipment, new technology to support remote care) and effective and timely support from health authorities.

*At the very least funding must be clear, transparent and predictable. (Female, 41 years old, family physician, CPHC, professional director)*

*The organisation of the primary care should not be left to each region or health centre separately but should be run by health authorities. (Female, 52 years old, nurse, CPHC, head of a unit)*

*It would make sense to upgrade the patients’ care system with appropriate technology that would allow better remote treatment, which would also be safe from GDPR aspect. (Male, 53 years old, family physician, private, head of department)*

*Our country should have a single plan for health care reorganisation in a health crisis. (Female, 41 years old, family physician, CPHC, professional director)*

## Discussion

### Main findings

In the category Information/instructions from decision makers, participants reported that instructions/guidelines were not clear and timely and were a major administrative burden. In the category of Organisation of work, participants indicated that the family medicine practices/health centres needed to organise themselves to be effective. This was done by reorganising care by establishing entry points for COVID patients, practices for acute and chronic patients (non-COVID), and administrative practices. In the Workforce category, participants reported staffing and equipment shortages and additional administrative burdens. In the PPE category, participants reported shortages of PPE, particularly in private family medicine practices. Participants also pointed to the lack of support for obtaining protective equipment from MH. The Views on decision-making Institutions category revealed that participants found SMC useful in terms of technical support. In contrast, support from NIPH and MH was perceived as slow and unclear. The category of Stressors that place additional burden on health care workers included uncertainty, anxiety, lack of support, high patient demands and rapidly changing work instructions.

In the Suggestions for improvement category, participants suggested clear work organisation in primary care and effective and timely support from health authorities.

### Comparison to other studies

#### Information/instructions from the decision-makers

Other studies (as well as our study) reported that primary care workers were overwhelmed with instructions from health authorities but lacked clear instructions [9,10]. In addition, our study found that the problem also lay in the timeliness, accuracy, and clarity of instructions, as well as the fact that this created an additional administrative burden.

#### Organisation of work

Ferencina et al. described an organisational model for primary care based on separate practices: for COVID, for non-COVID, and for administrative patients [11], which was also the case in our study. Other studies reported various organisational adjustments to separate infectious from non-infectious patients [4]. Mainly, this occurred at the level of individual practices [12]. In Slovenia, this occurred at the level of PCHCs and/or an administrative region level [13]. This could be a safeguard against the delayed care that occurred in primary care during the pandemic of COVID-19 and has been suggested in other studies [4,9,10,12].

The present study pointed out that primary care relied on its own abilities to organise during the pandemic. This has also been noted in other studies [14,15].

#### Workforce

Because of the increased need for health services, there was a shortage of suitable personnel, both doctors and nurses and administrative staff. This was evident in some other countries but not all [3]. In Slovenia, there has been a long-standing shortage of primary care professionals (especially physicians and nurses) [16]. Thus, this problem was not unexpected. Enrolled participants also pointed out that staff lacked additional training, which has not yet been identified in existing studies.

#### Personal protective equipment

Lack of PPE was a major problem at the beginning of the pandemic. This was a common problem in almost all countries [12,17–19].

#### Views on decision-making institutions

Views on decision-making institutions were often hostile. In other countries, dissatisfaction with the work of decision-making bodies, organisation and financing was lower [7,19]. In Slovenia, the health care system (especially organisation and financing) needs fundamental reform to adapt to existing and future challenges [16]. The negative views of the participants were the result.

#### Stressors that place an additional burden on healthcare workers

Physicians were on the front line during the pandemic, and there were many new situations and challenges related to the organisation and adaptation of primary care services [20–22]. The study conducted in France also showed that GP was under occupational stress during the pandemic, with women being more stressed [23]. A systematic review showed negative effects of the pandemic on the well-being of family physicians [24]. Slovenia was no exception.

#### Suggestion for improvements

Based on participants’ experiences and recommendations, the most important areas to address in similar pandemic situations are a clear organisation of work in primary care (adequate funding, staff allocation, distribution of personal protective equipment), psychological solid support for health workers, and effective and timely support from health authorities.

### Strengths and limitations

This was the first study on this topic in Slovenia and one of the few in Europe [3,4,12,19].

A limitation of this study is the method of data collection. Probably focus groups would be better suited to explore the topic in more depth and allow for stimulation between participants. Data collection was conducted when the first wave of the pandemic in Slovenia was still ongoing. Therefore, we considered an online questionnaire with open-ended questions to be the most feasible method for this study.

Another limitation is the inclusion of eight of the ten administrative health regions. This could affect the data quality because some regional characteristics may not have been included.

Barriers to accessibility for vulnerable groups have been identified in some other studies [25]. People with disabilities, mental health problems, migrants, homeless people, etcetera already live conditions that have been exacerbated by the pandemic. In our study, the questions about vulnerable groups were not specified, so we could not investigate our participants’ experiences with this issue.

### What does this study add?

This study provided new insights into the organisation of primary care during a pandemic in a country with strong public primary care through CPHCs [26]. Although health authorities lacked direction, primary care organised itself to treat all patients, including COVID [27–29]. This was done through a centralised approach with swab sites and separate practices for infectious and non-infectious patients. There was a shortage of staff. Psychological support was readily available.

## Conclusion

Based on participants’ experiences and suggestions, the most critical areas to address in similar pandemic situations are a clear organisation of work in primary care (adequate funding, staff allocation, distribution of personal protective equipment), strong psychological support for health workers and effective and timely support from health authorities.
